# Wnt Signaling and Circular RNAs in Esophageal and Gastric Cancers: Opportunities for Early Detection and Targeted Therapy

**DOI:** 10.3390/jcm14134805

**Published:** 2025-07-07

**Authors:** Piotr Paweł Chmielewski, Bartłomiej Strzelec, Julia Rudno-Rudzińska

**Affiliations:** 1 Division of Anatomy, Department of Human Morphology and Embryology, Faculty of Medicine, Wroclaw Medical University, 6a Chalubinskiego Street, 50-368 Wroclaw, Poland; 2 2nd Department of General Surgery and Surgical Oncology, Wroclaw Medical University, Borowska 213, 50-556 Wroclaw, Poland; bstrzelec94@interia.pl (B.S.); julia.rudno-rudzinska@umw.edu.pl (J.R.-R.); 3 University Center of General and Oncologic Surgery, Wroclaw Medical University, 50-556 Wroclaw, Poland

**Keywords:** circular RNAs (circRNAs), esophageal and gastric cancer, gastrointestinal cancers, liquid biopsy biomarkers, oncogenic signaling networks, Wnt signaling, precision oncology

## Abstract

Aberrant activation of Wnt/β-catenin signaling, frequently caused by oncogenic mutations, plays a crucial role in the development, progression, and therapy resistance of gastric, esophageal, hepatic, pancreatic, and colorectal cancers. Concurrently, circular RNAs (circRNAs), produced by back-splicing of precursor mRNAs (pre-mRNAs), have emerged as critical modulators of this pathway. Accumulating evidence indicates that specific circRNAs regulate Wnt/β-catenin signaling by sponging microRNAs, interacting with RNA-binding proteins, modulating protein function, and altering the expression of pathway components. Some circRNAs are also subject to feedback regulation by Wnt signaling itself. Clinically, tumor-associated circRNAs are present in body fluids and correlate with disease stage, metastatic burden, and patient survival, underscoring their potential as early and minimally invasive biomarkers. Moreover, targeting oncogenic circRNAs has shown promise in preclinical models of Wnt-driven gastrointestinal malignancies. In this review, we summarize the current understanding of the interplay between circRNAs and Wnt/β-catenin signaling in gastric and esophageal cancers. We discuss the translational challenges and emerging opportunities for biomarker development and targeted therapy.

## 1. Introduction

Esophageal and gastric cancers are among the deadliest malignancies of the digestive tract, developing insidiously with few or no early symptoms [1,2,3,4,5]. As a result, diagnosis is frequently delayed until advanced stages, when disease progression is highly aggressive, therapeutic options are limited, and overall survival (OS) for patients with distant metastases often falls below 5%. The pronounced molecular heterogeneity of these tumors adds another layer of complexity, complicating both diagnosis and treatment and contributing to poor clinical outcomes.

Although serum biomarkers such as carcinoembryonic antigen (CEA) and carbohydrate antigen 19-9 (CA19-9) have shown promise, and targeted therapies including human epidermal growth factor receptor 2 (HER2) inhibitors, anti-angiogenic compounds, and immune checkpoint inhibitors have improved outcomes in selected patients [6,7,8,9,10,11], early detection and effective treatment remain out of reach for most. Consequently, the management of gastric and esophageal cancers remains one of the most challenging areas in current oncology [2,3,4]. Identifying reliable biomarkers and effective molecular targets is therefore a critical priority. Recent studies have identified promising candidates such as claudin 18.2 (CLDN18.2) and fibroblast growth factor receptor 2 (FGFR2), both currently under clinical investigation [12,13,14,15]. Meanwhile, non-invasive approaches using circulating cell-free DNA (cfDNA) are emerging as valuable tools for early detection, treatment selection, and monitoring drug resistance [16]. These developments reflect a shift towards precision oncology, where treatment decisions are based on the molecular profile of a given tumor.

Within this evolving framework, circular RNAs (circRNAs) have emerged as a novel class of regulatory molecules with substantial clinical and biological relevance [4,5,6]. CircRNAs are covalently closed, single-stranded RNA molecules characterized by high structural stability and resistance to exoribonuclease degradation, which facilitates their detection in tissues and bodily fluids, including blood and saliva. CircRNAs regulate gene expression through diverse mechanisms, including microRNA (miRNA) sponging, modulation of RNA-binding protein interactions, and control of transcription and translation [5,6,17,18,19,20,21,22,23,24,25,26,27,28,29,30,31]. Their dysregulation has been implicated in multiple oncogenic processes, including uncontrolled proliferation, invasiveness, metastasis, and therapeutic resistance.

In gastrointestinal cancers, circRNAs have been increasingly recognized as key regulators of major signaling pathways involved in tumor progression, including Wnt/β-catenin signaling, which is an evolutionarily conserved pathway that regulates cell proliferation, epithelial–mesenchymal transition (EMT), and resistance to standard therapies [1,2,3,4,5,6]. Aberrant activation of this pathway constitutes a hallmark of a substantial subset of gastric and esophageal tumors. A growing body of evidence indicates that circRNAs affect Wnt/β-catenin signaling by sequestering inhibitory miRNAs, stabilizing oncogenic transcripts, and modulating downstream effector expression, thereby promoting malignant progression and resistance to therapy [4]. Despite these advances, several critical questions remain unresolved. How do circRNA-mediated regulatory networks influence therapeutic response or resistance to chemotherapy, immunotherapy, and targeted agents across diverse clinical settings? Can this knowledge be translated into clinically actionable diagnostic, prognostic, and therapeutic strategies to improve outcomes in patients with gastroesophageal malignancies?

In this narrative review, we explore the interplay between circRNAs and Wnt/β-catenin signaling in esophageal and gastric cancers. We integrate emerging evidence to elucidate the molecular mechanisms underlying this crosstalk, evaluate its roles in tumor progression and treatment resistance, and identify key gaps that warrant further investigation to inform the development of early diagnostic tools and targeted therapies for gastrointestinal cancers.

## 2. Circular RNAs: Biology, Formation, and Function

A particularly promising area of RNA research is the study of circular RNAs (circRNAs). These covalently closed, single-stranded transcripts lack 5′ and 3′ ends and poly(A) tails, rendering them resistant to exoribonuclease-mediated degradation such as RNase R [5,6]. This unique structure imparts exceptional stability and allows their detection in various body fluids, including serum and plasma, which positions circRNAs as promising candidates for non-invasive cancer diagnostics [18,25,29].

**Figure 1:**
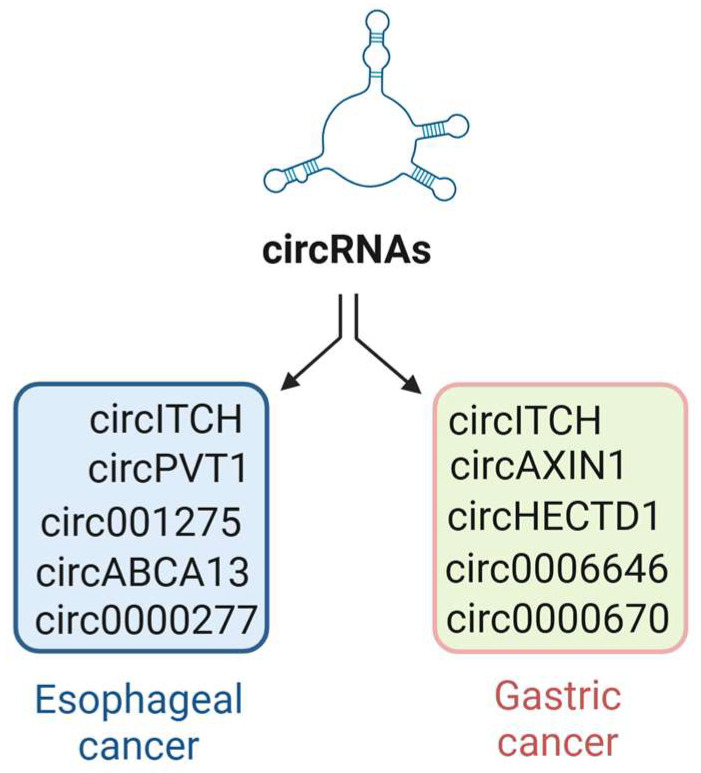
**CircRNAs regulate gene expression in esophageal and gastric cancers.** CircRNAs have been shown to act as miRNA sponges, modulating the critical Wnt/β-catenin signaling pathway that drives tumorigenesis. They also interact with RNA-binding proteins to control protein activity and localization and function as scaffolds for protein complexes.

Initially considered splicing by-products without functional significance, circRNAs have recently emerged as important regulators of gene expression, protein function, and cellular processes [4,5,6,24,25,26,27,28,29,30,31]. Advances in high-throughput sequencing and bioinformatics have led to the identification of numerous differentially expressed circRNAs across cancer types, including gastric and esophageal malignancies (Figure 1), supporting their utility as biomarkers and therapeutic targets [4,5].

CircRNAs are derived from canonical pre-mRNA transcripts via a non-canonical splicing event known as back-splicing. In this process, a downstream 5′ splice-donor site joins to an upstream 3′ splice-acceptor site, forming a closed-loop RNA molecule through a 3,5′-phosphodiester bond. CircRNAs are typically composed of one to five exons and localize predominantly to the cytoplasm, although some intron-containing circRNAs are retained in the nucleus and exert transcriptional regulatory functions.

Four mechanisms of circRNA biogenesis have been identified [5,6,18]: (1) RNA-binding protein (RBP)-driven cyclization, in which RBPs facilitate circularization by bringing flanking introns into proximity; (2) intron pairing-driven cyclization, mediated by complementary sequences such as Alu elements; (3) exon-skipping or lariat-driven cyclization, involving lariat intermediates stabilized by factors including U2AF65 and SF1; and (4) lariat intron-driven cyclization, whereby circular intronic RNAs (ciRNAs) are formed through specific sequence motifs that evade debranching enzyme degradation. These mechanisms underscore the complexity of circRNA biosynthesis and its tight regulation.

Functionally, circRNAs participate in a range of cellular activities. They act as competitive endogenous RNAs (ceRNAs), sequestering microRNAs (miRNAs) through conserved binding sites and thereby mitigating miRNA-mediated repression of target mRNAs [30,31]. This activity is particularly relevant to cancer, where miRNAs influence key processes such as chemosensitivity and immune evasion [19]. CircRNAs also bind to RBPs, modulate transcription and translation, and serve as scaffolds for protein complexes. Although most circRNAs are non-coding RNAs (ncRNAs), some harbor internal ribosome entry sites (IRESs) or N6-methyladenosine (m6A) modifications, allowing for cap-independent translation [5,6].

CircRNAs are involved in the regulation of classical oncogenic pathways, including Wnt, MAPK, Hedgehog, and c-Myc. Their expression and functional relevance have been documented in various digestive system malignancies, including gastric and esophageal cancers. Furthermore, circRNAs are enriched in exosomes, mediating intercellular communication that promotes tumor growth, invasion, and metastasis [18,23]. The continued refinement of experimental standards and methodologies for circRNA isolation and analysis will be essential for translating their biological potential into clinical application. As evidence accumulates, circRNAs are increasingly regarded as clinically relevant biomarkers and potential therapeutic targets in oncology.

In recent years, circRNAs have gained increasing attention for their role in the pathogenesis of various cancers, including liver, pancreatic, colorectal, and gastroesophageal malignancies [4,5,6,22,23,24,25,26,27,28,29,30,31]. In particular, their interaction with pivotal signaling pathways, such as the Wnt pathway, has emerged as a major area of focus in molecular oncology [32,33,34,35,36,37]. The Wnt signaling cascade, which regulates key cellular processes like proliferation, differentiation, and apoptosis, is frequently dysregulated in cancers affecting the digestive system. Central to this dysregulation is β-catenin, which is a downstream effector of the Wnt pathway. Abnormal activation of β-catenin has been identified as a hallmark of tumorigenesis in esophageal, gastric, hepatic, pancreatic, and colorectal cancers, contributing to tumor progression, metastasis, and chemoresistance.

Building on this understanding, recent studies have demonstrated that circRNAs affect the activity of β-catenin and other components of the Wnt pathway [38]. This interaction suggests that circRNAs may play an essential role in regulating the oncogenic properties of β-catenin, potentially offering novel insights into tumor biology. The dysregulation of circRNAs in the context of Wnt/β-catenin signaling represents a promising avenue for cancer diagnosis, prognosis, and therapeutic intervention. The exploration of circRNAs as early biomarkers holds great promise, particularly for early detection and personalized treatment strategies [38].

In addition to their roles in cancer progression, circRNAs have also been implicated in tumor metastasis and chemoresistance. Recent studies have shown that circRNAs derived from the Plasmacytoma Variant Translocation 1 (*PVT1*) gene (circPVT1) regulate 5-fluorouracil (5-FU) chemosensitivity in esophageal squamous cell carcinoma (ESCC) by modulating the Wnt/β-catenin pathway through the miR-30a-5p/FZD3 axis [39]. In fact, circRNAs can enhance the invasive potential of cancer cells, promote angiogenesis, and contribute to resistance against conventional therapies, thanks to their ability to modulate key signaling pathways. The discovery of exosomal circRNAs, which are secreted into the extracellular space and can be detected in body fluids, further underscores their potential as new non-invasive biomarkers for monitoring cancer progression and therapeutic response [29,38,39].

A growing body of evidence highlights the multifaceted roles of circRNAs in cancer biology, particularly in gastrointestinal cancers and other malignancies affecting the digestive system [4,5,34,37,38]. Their involvement in key signaling pathways, such as Wnt/β-catenin, underscores their potential as both diagnostic and prognostic biomarkers, as well as therapeutic targets. Despite their promising potential, substantial challenges remain in elucidating the molecular mechanisms of circRNAs and translating these insights into effective clinical applications.

## 3. The Role of Wnt Signaling in Esophageal and Gastric Cancers

**Figure 2:**
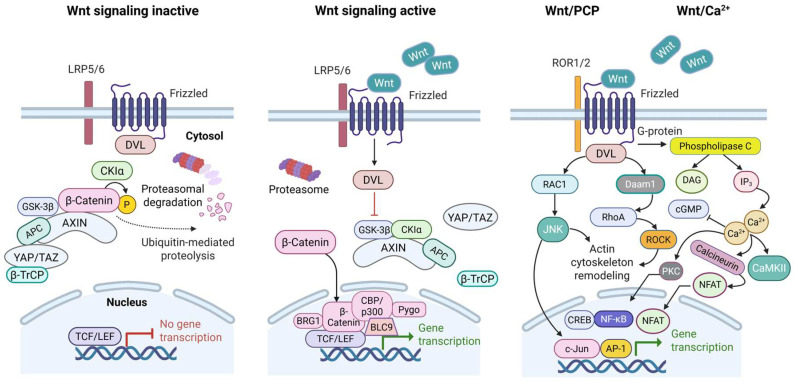
**Overview of Wnt Signaling Pathways:** canonical (Wnt/β-catenin) and non-canonical (Wnt/planar cell polarity, PCP and Wnt/Ca^2+^) pathways. In the absence of Wnt ligands, β-catenin is phosphorylated by the destruction complex, which consists of Axin, adenomatous polyposis coli (APC), glycogen synthase kinase 3β (GSK3β), and casein kinase 1α (CK1α). Phosphorylated β-catenin is recognized by β-transducin repeat-containing proteins (β-TrCP), ubiquitinated and targeted for proteasomal degradation. As a result, Wnt target genes are repressed by TCF/LEF transcription factors complexed with corepressors such as TLE/Groucho. Upon Wnt ligand binding to Frizzled receptors and LRP5/6 co-receptors, LRP phosphorylation promotes the recruitment of Disheveled (DVL) to the plasma membrane. Activated DVL disrupts the destruction complex, preventing β-catenin degradation. Stabilized β-catenin accumulates in the cytoplasm and translocates into the nucleus, where it displaces TLE/Groucho from TCF/LEF, forming an active transcriptional complex. This complex, assisted by co-activators, including CBP/p300, BCL9, Pygo, and BRG1, induces the expression of Wnt target genes. Noncanonical Wnt signaling (Wnt/PCP and Wnt/Ca^2+^) activates DVL-associated activator of morphogenesis 1 (Daam1), RhoA–Rho-associated kinase (ROCK), and Rac1–c-Jun N-terminal kinase (JNK), inducing cytoskeletal remodeling. The Wnt/Ca^2+^ pathway signals via phospholipase C, diacylglycerol (DAG), inositol trisphosphate (IP_3_), and Ca^2+^, which inhibits cyclic guanosine monophosphate (cGMP) while activating protein kinase C (PKC), calmodulin-dependent kinase II (CaMKII), nuclear factor of activated T cells (NFAT), cyclic AMP response element-binding (CREB) protein, nuclear factor kappa B (NF-κB), and activator protein 1 (AP-1).

Wnt signaling is an evolutionarily conserved pathway that regulates cell proliferation, differentiation, migration, and survival [5,32,33,34,35,36,37,38]. It is activated by Wnt ligands binding to Frizzled (FZD) receptors at the cell surface, which initiates intracellular signaling cascades. Wnt signaling is broadly categorized into two branches: the canonical (β-catenin-dependent) and noncanonical (β-catenin-independent) pathways (Figure 2).

The canonical Wnt/β-catenin pathway is a key regulator of cellular proliferation, survival, and differentiation, and it plays a central role in tumorigenesis, including in gastric and esophageal cancers [5,32,33,34,37,38]. This pathway is initiated when Wnt ligands engage FZD receptors and the co-receptors LRP5 or LRP6 at the plasma membrane [5,34]. In the absence of Wnt ligands, cytoplasmic β-catenin is continuously degraded by a multiprotein destruction complex composed of the tumor suppressors adenomatous polyposis coli (APC) and Axin, as well as glycogen synthase kinase 3 (GSK3), casein kinase 1α (CK1α), and protein phosphatase 2A (PP2A). CK1α and GSK3 sequentially phosphorylate β-catenin at conserved N-terminal residues (Ser45, Thr41, Ser37, and Ser33), targeting it for β-TrCP-mediated ubiquitination and proteasomal degradation [34].

Upon Wnt ligand binding, the FZD–LRP5/6 complex recruits the cytoplasmic scaffold protein Disheveled (DVL), which becomes phosphorylated and oligomerizes [5,6,34]. This facilitates the recruitment of Axin to the phosphorylated cytoplasmic tail of LRP5/6. Axin binding is stabilized by GSK3- and CK1γ-mediated phosphorylation of LRP5/6, which sequesters Axin at the membrane, disrupts the destruction complex, and inhibits β-catenin phosphorylation [34]. Stabilized β-catenin accumulates in the cytoplasm and translocates into the nucleus. In the nucleus, β-catenin displaces co-repressors from T-cell factor/lymphoid enhancer-binding factor (TCF/LEF) transcription factors and recruits co-activators such as CBP/p300, BCL9, Pygopus, and others to form an active transcriptional complex [5,28,32,34]. This complex induces the expression of Wnt target genes involved in cell cycle progression (e.g., *MYC* and *CCND1*), survival (e.g., *BIRC5*), epithelial-to-mesenchymal transition (e.g., *ZEB1*, *SNAI1*), and stemness (e.g., *LGR5* and *SOX9*). The composition of β-catenin transcriptional complexes is cell-type specific and regulated by context-dependent co-factors, conferring spatial and temporal specificity to Wnt target gene expression.

**Figure 3:**
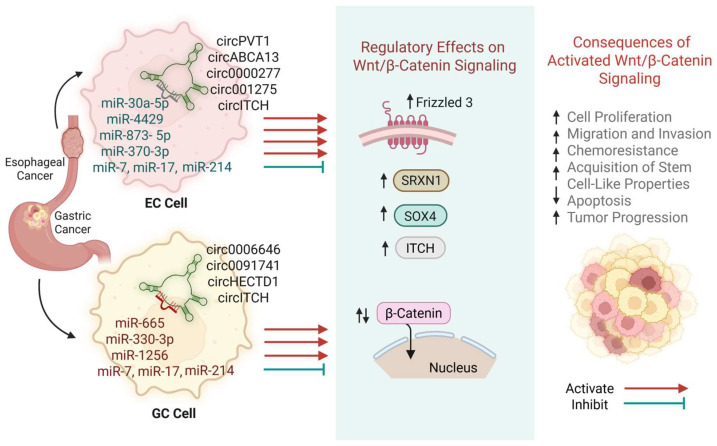
**In esophageal and gastric cancers (EC and GC, respectively), circRNAs modulate Wnt/β-catenin signaling through various mechanisms. CircRNAs act as sponges for miRNAs.** They also interact with RNA-binding proteins and engage in other regulatory processes to upregulate Wnt pathway components including *SRXN1*, *SOX4*, *FZD3*, and *WNT7A*. This enhances β-catenin stabilization and nuclear translocation, promoting malignant phenotypes such as proliferation, migration, invasion, chemoresistance, and acquisition of stem-like properties. Conversely, tumor-suppressive circRNAs, such as circITCH, function as molecular sponges for miR-7, miR-17, and miR-214 and increase the expression of *ITCH*, which results in the inhibition of Wnt/β-catenin signaling and constrains tumor progression.

Additional regulatory layers modulate β-catenin activity. For example, β-catenin can be phosphorylated at Ser552 by protein kinase B (Akt), which reduces its affinity for cadherin complexes and promotes cytoplasmic accumulation and nuclear import. Nuclear translocation is further facilitated by interaction with 14-3-3ζ, which binds phosphorylated β-catenin and assists its nuclear localization. High-throughput proteomic studies have revealed a dynamic and extensive β-catenin interactome, indicating that its transcriptional activity is finely tuned through multiple protein–protein interactions across distinct oncogenic contexts. Aberrant activation of the Wnt/β-catenin pathway is a hallmark of both gastric and esophageal cancers [37,38] (Figure 3).

In gastric cancer, somatic mutations in *CTNNB1* (encoding β-catenin), as well as inactivation of negative regulators such as *APC* and *AXIN1*, lead to constitutive β-catenin stabilization, nuclear accumulation, and persistent activation of Wnt target genes [4,34,37,38]. In esophageal squamous cell carcinoma (ESCC), nuclear β-catenin correlates with poor prognosis, advanced tumor stage, and chemoradiotherapy resistance. Overexpression of Wnt ligands (e.g., WNT1, WNT7A) and FZD receptors has been observed in both gastric and esophageal adenocarcinomas, where it contributes to epithelial-to-mesenchymal transition (EMT), immune evasion, and metastasis.

Circular RNAs (circRNAs) regulate Wnt/β-catenin signaling at multiple levels [4,5,6,22,24,25,26,27,28]. One of the principal mechanisms involves their function as sponges for Wnt-inhibitory microRNAs (miRNAs), thereby relieving repression of Wnt pathway components. Oncogenic circRNAs promote the expression of Wnt activators such as *SOX4*, *SRXN1*, *FZD3*, and *WNT7A* by sequestering miRNAs that target these transcripts. This facilitates β-catenin stabilization and nuclear translocation, amplifying downstream transcriptional programs that drive proliferation, migration, invasion, chemoresistance, and stemness.

In contrast, tumor-suppressive circRNAs such as circITCH inhibit Wnt signaling by acting as molecular sponges for miR-7, miR-17, and miR-214 [37,38]. These miRNAs suppress the expression of ITCH, an E3 ubiquitin ligase that targets DVL for degradation. By enhancing ITCH levels, circITCH promotes DVL degradation, thereby inhibiting canonical Wnt activation and attenuating tumor progression. Loss of tumor-suppressive circRNAs has been associated with hyperactivation of β-catenin signaling and more aggressive disease in both gastric and esophageal cancers [38]. These findings underscore the role of circRNAs as upstream regulators of oncogenic Wnt signaling. The noncanonical Wnt pathways are subdivided into the Wnt/planar cell polarity (PCP) pathway, also referred to as the Wnt/c-Jun N-terminal kinase (JNK) pathway, and the Wnt/calcium (Wnt/Ca^2+^) pathway (Figure 2). These pathways do not rely on β-catenin and instead regulate cell polarity, motility, cytoskeletal architecture, and adhesion [5,34]. Their dysregulation has been implicated in invasion, metastasis, and treatment resistance in gastrointestinal malignancies.

The Wnt/PCP pathway is initiated by the binding of Wnt ligands to FZD receptors and co-receptors such as receptor tyrosine kinase-like orphan receptors 1 and 2 (ROR1/ROR2) or protein tyrosine kinase 7 (PTK7) [5]. This activates DVL, which recruits small GTPases including RhoA and Rac1. RhoA activates Rho-associated coiled-coil-containing protein kinase (ROCK), leading to actomyosin contractility and regulation of cell polarity. Concurrently, Rac1 activates JNK, which orchestrates actin cytoskeletal remodeling and supports directional cell migration. These processes promote cell motility and invasiveness, particularly during EMT.

The Wnt/Ca^2+^ pathway also begins with Wnt–FZD engagement but signals through trimeric G-proteins that activate phospholipase C (PLC) [5,34]. PLC hydrolyzes phosphatidylinositol 4,5-bisphosphate (PIP_2_) to produce diacylglycerol (DAG) and inositol 1,4,5-trisphosphate (IP_3_). IP_3_ binds to receptors on the endoplasmic reticulum, inducing Ca^2+^ release into the cytosol. Increased cytosolic Ca^2+^ activates downstream effectors including protein kinase C (PKC), calmodulin-dependent protein kinase II (CaMKII), and calcineurin (Figure 2). Calcineurin dephosphorylates and activates nuclear factor of activated T cells (NFAT), a transcription factor that controls genes involved in cell adhesion, migration, and immune regulation. CaMKII and Ca^2+^-dependent pathways also activate cyclic AMP response element-binding protein (CREB) and nuclear factor kappa-light-chain-enhancer of activated B cells (NF-κB), which promote cell survival, inflammation, and therapy resistance. These pathways contribute to invasive phenotypes and resistance to cytotoxic stress in gastric and esophageal tumors.

Importantly, both Wnt/PCP and Wnt/Ca^2+^ pathways intersect with the canonical pathway [5,34,38]. For example, CaMKII and NFAT can antagonize β-catenin signaling by interfering with Wnt target gene transcription. This crosstalk adds complexity to Wnt-driven oncogenesis and promotes phenotypic plasticity in tumor cells. The noncanonical Wnt pathways are increasingly recognized as important contributors to the malignant progression of gastroesophageal cancers. Although targeting these pathways holds therapeutic promise, their context-dependent roles and integration with canonical signaling complicate the development of effective Wnt-targeted therapies.

### 3.1. Esophageal Cancer

Wnt signaling is crucial in the pathogenesis of esophageal cancer as well as other malignancies, where it regulates key processes such as tumor cell proliferation, migration, and invasion [5,32,33,34,35,36,37,38], and is also modulated by circRNAs such as circABCA13, circ0000277, circPVT1, circ001275, and circITCH [38]. Dysregulation of this pathway often leads to the stabilization and nuclear translocation of β-catenin, which is a protein encoded by the *CTNNB1* gene [5], whose mutations or overexpression are associated with several cancers. β-catenin acts as a key transcriptional coactivator, driving the expression of genes involved in cell growth and survival.

Currently, several circRNAs have been identified as key modulators of the Wnt pathway, influencing esophageal cancer progression and response to chemotherapy. For instance, circABCA13 and circ0000277 are significantly upregulated in ESCC tissues, and their expression correlates with lymph node metastasis and advanced tumor stages [5,22,24,25,26,27,28,38]. It has been established that depletion of circABCA13 impairs cell proliferation and migration, while silencing circ0000277 curtails cell proliferation and promotes apoptosis. In particular, this knockdown hinders the transition from the G0/G1 phase to the S phase, thus halting the cell cycle [38,40]. These findings suggest that circRNAs linked to the Wnt/β-catenin pathway can serve not only as valuable biomarkers but also as potential therapeutic targets.

CircABCA13 functions as a sponge for miR-4429, leading to increased *SRXN1* expression and subsequent activation of the Wnt/β-catenin pathway, thus promoting ESCC cell proliferation, migration, and invasion [41]. Circ0000277 sequesters miR-873-5p, thus elevating *SOX4* expression and further activating Wnt signaling, which contributes to tumor progression [40]. Similarly, circPVT1 acts as a sponge for miR-30a-5p, thus regulating chemosensitivity through the miR-30a-5p/FZD3 axis while also modulating Wnt signaling and ferroptosis in ESCC [38,39].

In contrast, circRNA itchy E3 ubiquitin protein ligase (circITCH) inhibits the Wnt pathway by sequestering several miRNAs, including miR-7, miR-17, and miR-214, leading to increased *ITCH* expression, which in turn negatively regulates the Wnt pathway [22,34]. Downregulation of circITCH promotes ESCC cell proliferation and inhibits apoptosis, highlighting its potential as a therapeutic target for inhibiting Wnt signaling.

Overall, these findings reveal an important role for circRNAs in modulating the Wnt/β-catenin pathway. By acting as sponges for specific miRNAs, circRNAs regulate the expression of critical genes involved in tumorigenesis, chemoresistance, and metastasis [5,6,22,24,27,28,29,38,41,42,43]. The increasing evidence of circRNAs as non-invasive markers for early cancer detection, prognosis, and treatment response further support their clinical potential [28,29]. Targeting these circRNAs offers a promising avenue for therapeutic intervention, particularly in inhibiting tumor progression and overcoming its chemoresistance.

### 3.2. Gastric Cancer

Gastric cancer represents a significant global health challenge, characterized by high mortality rates and aggressive progression, often leading to poor prognosis and limited therapeutic options [5,6,9]. Despite substantial advances in diagnostic techniques and treatment strategies, the overall survival rate for gastric cancer remains exceptionally low, underscoring the urgent need for a deeper understanding of its molecular mechanisms to inform the development of more effective therapies.

Among the key signaling pathways implicated in gastric cancer progression, the Wnt/β-catenin pathway stands out as a critical regulator of cellular processes, including proliferation, differentiation, and metastasis. Recent studies have shown that circRNAs play a pivotal role in modulating Wnt signaling and contributing to the pathogenesis of gastric cancer. Currently, several circRNAs have been identified as key contributors to the development of gastric cancer. For example, circAXIN1, circHECTD1, circ0006646, circ0000670, as well as circ0091741 are significantly upregulated in gastric cancer tissues and cell lines (see Figure 1), correlating with advanced disease stages, poor prognosis, and key malignant traits [38].

For example, recent studies have reported that circAXIN1 is highly expressed in gastric cancer tissues compared to paired adjacent normal gastric tissues. Moreover, elevated circAXIN1 levels correlate with deeper tumor invasion and lymph node metastasis, suggesting that it may serve as a candidate prognostic biomarker, pending further validation. In addition, circAXIN1 encodes a 295-amino-acid protein, AXIN1-295aa, which appears to activate the Wnt signaling pathway (see Figure 2), thereby promoting tumorigenesis and the progression of gastric cancer [42]. Mechanistically, AXIN1-295aa harbors a regulator of G-protein signaling (RGS) domain that enables it to interact with a 25-amino-acid SAMP motif from APC. As a result, AXIN1-295aa competes with AXIN1 for binding to APC, disrupting the recruitment of key components of the β-catenin destruction complex, including APC and GSK3β. This interference prevents AXIN1, CK1α, and GSK3β from forming a destruction complex, which is essential for β-catenin phosphorylation and subsequent proteasomal degradation. Consequently, β-catenin accumulates in the cytoplasm and translocates into the nucleus, where it activates downstream Wnt target genes that drive gastric cancer cell proliferation and migration. Given the central role of dysregulated Wnt signaling in oncogenesis [32,33,34,37,38], the ability of AXIN1-295aa to disrupt β-catenin turnover and sustain pathway activation suggests that it may significantly contribute to tumorigenesis and disease progression in gastric cancer.

Recent studies have also shown that higher levels of circ0006646 expression are correlated with advanced TNM stage and lymph node invasion, with Kaplan–Meier analysis indicating an association between elevated circ0006646 levels and shorter overall survival in gastric cancer patients. In addition, circHECTD1 influences the aggressiveness of gastric cancer by modulating glutaminolysis, potentially affecting key enzymes and metabolite levels in tumor cells [38]. Furthermore, circ0091741 has been implicated in enhancing cell migration and invasion through its interaction with miR-330-3p, which appears to increase *TRIM14* expression, potentially stabilizing DVL2 and activating the Wnt/β-catenin pathway, although the precise impact of this regulatory axis remains to be fully elucidated.

The therapeutic potential of targeting circRNAs in gastric cancer appears particularly promising. CircRNAs not only influence signal transduction pathways and cellular metabolism, but they also function as miRNA sponges, thereby affecting gene expression and treatment outcomes, including chemoresistance [41,42,43]. Moreover, several studies have demonstrated that suppressing circ0000670 and circ0091741 inhibits cancer cell growth in vivo [38]. Furthermore, administering siRNAs targeting these circRNAs has been shown to suppress metastasis in animal models, further supporting their viability as therapeutic targets. Conversely, the overexpression of circITCH suppresses cell proliferation, migration, and invasion by attenuating β-catenin signaling.

Beyond their interactions with miRNAs, circRNAs also modulate key components of the Wnt signaling pathway. For example, circ0091741 is proposed to affect the Wnt/β-catenin cascade through modulation of *TRIM14* and stabilization of DVL2, whereas circAXIN1, derived from a gene that negatively regulates Wnt signaling, is associated with enhanced cell proliferation and migration via its effects on pathway components. Furthermore, circITCH functions as a sponge for miRNAs (e.g., miR-17), leading to upregulation of its linear counterpart, *ITCH*, which may promote the degradation of DVL2 and subsequently inhibit β-catenin activation.

Although these findings underscore the critical role of circRNAs in regulating the Wnt pathway, further research is required to unravel the multiplicity of mechanisms and their clinical implications. Exosomal circRNAs have been shown to facilitate cancer progression and metastasis, reinforcing their potential as non-invasive biomarkers for early detection and monitoring of therapeutic responses, given that they are secreted into the extracellular space and can be readily detected in body fluids. Moreover, the inhibitory effects of circRNAs on the Wnt pathway further highlight their therapeutic promise. Consequently, targeting circRNAs may offer promising strategies for enhancing the management of gastric cancer in the future.

## 4. Clinical Implications, Challenges, and Future Directions

Aberrations in the Wnt/β-catenin pathway contribute significantly to the pathogenesis of gastrointestinal cancers [36,38,40,44,45,46,47,48]. Due to its central role in regulating cellular proliferation, differentiation, and migration, the Wnt/β-catenin axis, together with circRNAs, has emerged as a target for therapeutic intervention [29,38,49,50,51,52,53] (Table 1).

In esophagogastric cancers, dysregulation of the Wnt/β-catenin pathway drives oncogenic transformation, modulates the tumor microenvironment (TME), and alters host immune responses [54,55,56,57]. Activation of this pathway results in cytoplasmic accumulation and nuclear translocation of β-catenin, which interacts with TCF/LEF transcription factors to promote the expression of genes involved in cell survival, proliferation, and invasion (Figure 2) [32,33,38,50,52]. These events contribute to tumor progression and metastasis, including both lymphatic and distant dissemination [55,56]. Elevated levels of nuclear and cytoplasmic β-catenin, detected by immunohistochemistry and Western blotting, serve as indicators of pathway activation and are associated with poor clinical outcomes in gastric cancer [4,54].

In esophageal cancer, aberrant Wnt signaling promotes tumorigenesis, remodels the TME, and contributes to therapeutic resistance [57,58,59]. Preclinical studies suggest that targeted modulation of this pathway can enhance immunotherapy efficacy, particularly when combined with monoclonal antibodies [49,53,57,58,59,60]. Such combinatorial approaches may overcome resistance mechanisms and improve clinical outcomes in patients with gastrointestinal cancers.

The Wnt/β-catenin pathway intersects with other key signaling cascades, including MEK-ERK, PI3K, and NOTCH [34,37]. Therefore, therapeutic strategies must be highly specific to minimize off-target effects and systemic toxicity. The development of selective inhibitors that modulate Wnt signaling without disrupting normal tissue homeostasis remains a significant challenge [61]. Furthermore, the interplay between prostaglandin E2 (PGE2) and Wnt signaling implicates chronic inflammation as a potential activator of this pathway, linking inflammatory states, aging, and carcinogenesis [59,62].

Ongoing clinical trials highlight the critical need for robust biomarkers to stratify patients and guide therapeutic decision making [50]. Personalized treatment strategies based on individual molecular profiles are essential for optimizing clinical outcomes [63,64]. Recent progress in Wnt pathway inhibitors, including monoclonal antibodies and antibody–drug conjugates (ADCs), aligns with the broader paradigm of precision oncology [65,66,67,68]. Concurrently, gene therapy approaches, such as antisense oligonucleotides (ASOs) and RNA interference (RNAi), are currently under intensive investigation. ASOs are short, single-stranded oligonucleotides designed to bind complementary mRNA sequences, leading to RNase H-mediated degradation or translational inhibition. In contrast, RNAi mediates post-transcriptional gene silencing through small interfering RNAs (siRNAs) or short hairpin RNAs (shRNAs).

Several natural compounds, including curcumin and polyphenols, have also shown the potential to modulate Wnt/β-catenin signaling in preclinical models [69,70,71,72]. Given the complexity of this pathway, monotherapies are unlikely to produce durable and meaningful clinical benefits. Consequently, combination regimens that integrate Wnt inhibitors with chemotherapy or immunotherapy are under active investigation, even though further studies are required to determine their safety and efficacy [73].

Recent advances have elucidated the interactions between circRNAs and Wnt/β-catenin signaling in a wide range of neoplasms, including gastrointestinal malignancies [5,6,34,36,38,40,47,74,75,76,77,78]. Targeting downstream effectors, such as the interaction between β-catenin and its co-activator CREB-binding protein (CBP), represents a promising therapeutic strategy for cancer treatment. For example, PRI-724 disrupts the β-catenin/CBP complex and selectively suppresses Wnt-driven transcription in preclinical models of gastric cancer. Inhibitors of porcupine O-acyltransferase (PORCN), including WNT974, have shown efficacy in preclinical models of esophageal adenocarcinoma, and their clinical evaluation in combination regimens is ongoing [49,50,51,52].

The therapeutic potential of targeting circRNAs has recently been supported by preclinical studies [42,43]. For example, ASOs designed to target circ-APC may restore repression of the Wnt pathway. Conversely, tumor-suppressive circRNAs such as circ-CTNNB1 inhibit Wnt signaling by sequestering miR-520h, which targets APC, which is a key negative regulator of β-catenin. Delivery of synthetic mimics of such tumor-suppressive circRNAs offers a specific approach to modulate Wnt activity in malignant cells. The interplay among Wnt signaling, circRNAs, and the TME reveals additional therapeutic vulnerabilities. Cancer-associated fibroblasts (CAFs) within the TME secrete Wnt ligands that promote cancer stemness and therapeutic resistance. Inhibition of Wnt ligand secretion using PORCN inhibitors is being explored to sensitize tumors to conventional and immune-based therapies. Furthermore, tumor-derived exosomal circRNAs modulate immune cell function, contributing to an immunosuppressive microenvironment. Disrupting this intercellular communication by inhibiting exosome release or targeting specific exosomal circRNAs might enhance immune checkpoint blockade efficacy [18,20].

To overcome challenges related to stability and delivery, advanced drug delivery platforms are in development. Nanoparticle-based systems, including ligand-functionalized liposomes, are engineered to selectively deliver Wnt inhibitors or nucleic acid therapeutics, such as siRNAs, ASOs, and circRNA mimics, to tumor tissues. This can improve therapeutic efficacy, reduce side effects, and enable personalized oncology approaches [38,50]. For example, liposome-encapsulated β-catenin inhibitors targeting receptors overexpressed on cancer cells are undergoing preclinical evaluation.

Despite these promising advances, significant translational challenges remain. Chief among these are the in vivo stability, delivery efficiency, and specificity of circRNA-targeting therapeutics, including ASOs and CRISPR-Cas systems. Naked nucleic acids are rapidly degraded and poorly internalized by cells. Lipid nanoparticles (LNPs), which have proven successful in mRNA vaccine platforms, are now being adapted to encapsulate and protect these agents. Additional delivery platforms under investigation include polymer-based nanoparticles, inorganic carriers such as gold nanoparticles, and engineered exosomes that offer low immunogenicity.

Mitigating off-target effects remains a critical challenge, particularly given the pleiotropic functions of many circRNAs, which interact with multiple microRNAs and RNA-binding proteins [4,5,6,17,19,38]. Indeed, achieving precise modulation requires comprehensive computational screening and rigorous experimental validation. Antisense oligonucleotides (ASOs) and CRISPR guide RNAs are being chemically optimized with high-affinity modifications, such as locked nucleic acids (LNAs), to enhance target specificity and minimize mismatch tolerance.

In summary, the Wnt/β-catenin signaling pathway and its regulatory circular RNAs (circRNAs) represent promising therapeutic targets in gastric and esophageal cancers [34,38]. Advances in molecular profiling, therapeutic development, and targeted delivery systems are enabling the creation of more precise and effective treatment strategies. However, translating these innovations into clinical practice will require thorough preclinical validation and carefully designed clinical trials to ensure their safety, efficacy, and long-term benefits for patients.

## 5. Conclusions

The Wnt/β-catenin signaling pathway plays a critical role in the development of gastric and esophageal cancers, as its aberrant activation promotes uncontrolled cell proliferation, invasion, metastasis, stemness, and resistance to therapy. From a clinical perspective, the identification of recurrent mutations in key pathway components, such as *CTNNB1*, *APC*, and *AXIN1*, has provided important insights into the molecular pathogenesis of gastrointestinal tumorigenesis. Moreover, growing evidence supports a bidirectional regulatory relationship between circRNAs and Wnt/β-catenin signaling. These circRNAs have direct implications for early detection, prognosis, and targeted therapy. Oncogenic circRNAs that enhance Wnt/β-catenin activity, as well as tumor-suppressive circRNAs that inhibit the pathway, are under investigation as promising biomarkers and potential therapeutic targets. The increasing recognition of circRNAs as important regulators of Wnt signaling presents novel opportunities for RNA-based therapies. Novel approaches may include synthetic circRNA mimics, ASOs, or RNA interference strategies to modulate circRNA–miRNA–mRNA regulatory networks. These strategies might offer greater specificity and lower systemic toxicity than conventional small-molecule inhibitors. However, challenges related to RNA delivery, molecular stability, and off-target effects must still be addressed. Future research should focus on identifying predictive biomarkers to guide patient selection and optimize Wnt-targeted therapies. Furthermore, integrated multi-omics profiling, including genomic, transcriptomic, and epigenetic analyses, will be essential. In addition, elucidating the interplay between Wnt signaling and the tumor microenvironment, including immune modulation and stromal remodeling, may inform rational combination strategies. For example, co-targeting Wnt and immune checkpoint pathways may help to overcome immune evasion in Wnt-driven tumors. In conclusion, Wnt/β-catenin signaling constitutes a critical oncogenic pathway in gastric and esophageal cancers. Although major advances have clarified its molecular architecture and functional significance, further research is needed to translate these insights into effective clinical applications. Continued progress will require coordinated efforts across molecular oncology, systems biology, and translational research. In the future, Wnt-targeted strategies may become essential components of precision oncology aimed at reducing the global burden of gastrointestinal malignancies.

## Figures and Tables

**Table 1:** Emerging therapeutic strategies targeting the Wnt/β-catenin pathway and circRNAs in esophageal and gastric cancers.

Cancer	Therapeutic Modality	Molecular Target	Main Outcomes	References
Esophageal	Monoclonal antibodies	FZD receptors	Reduced tumor growth in preclinical models	[49]
Esophageal and Gastric	RNA interference (siRNA/shRNA)	Key Wnt signaling components, e.g., β-catenin	Reduced Wnt pathway activity Decreased β-catenin levels Inhibition of tumor progression	[50]
Esophageal and Gastric	Small molecule inhibitors like AZ1366, LGK974, ETC-159	Tankyrase, Porcupine (PORCN)	Reduced tumor growth in preclinical models	[51,52]
Esophageal and Gastric	Circulating circRNAs as non-invasive biomarkers; RNAi	circRNAs	Early detection in patient blood samples Increased chemosensitivity Inhibited tumor growth	[29,38]
Gastric	Monoclonal antibodies such as Vantictumab (OMP-18R5)	FZD receptors	Reduced gastric adenoma growth in preclinical models	[53]

## Data Availability

Not applicable.
